# Re-examine the influence of organizational identification on unethical pro-supervisor behavior

**DOI:** 10.3389/fpsyg.2022.1060032

**Published:** 2022-12-22

**Authors:** Tuwei Sun, Wei Shi, Jing Wang

**Affiliations:** ^1^Chongqing City Branch, Industrial and Commercial Bank of China, Chongqing, China; ^2^School of Labor and Human Resources, Renmin University of China, Beijing, China; ^3^School of Management, Zunyi Medical University, Zunyi, China

**Keywords:** unethical pro-supervisor behavior, organizational identification, perceived organizational cronyism, felt obligation, the unethical behavior to benefit others

## Abstract

Employees’ unethical pro-supervisor behavior (UPSB) is common in organizations. Existing research primarily argued that organizational identification increases this behavior, emphasizing that UPSB benefits organizations indirectly. However, it ignores that UPSB can sometimes serve the interests of the supervisor at the expense of the interests of the organization. Drawing on social identity theory and social cognitive theory, this study aims to emphasize this point by proposing that organizational identification can inhibit employees’ UPSB *via* the mediation of felt obligation. We also propose that perceived organizational cronyism would weaken the negative effect. Data were collected through a self-reported online questionnaire based on a three-wave research design and analyzed through hierarchical regression analyses. With a sample of 578 Chinese employees, we found support for our propositions. Implications and limitations are discussed.

## 1. Introduction

Unethical pro-supervisor behavior (UPSB) refers to the type of work behavior that benefits supervisor by violating core social values, ethics, laws, or standards (Johnson and Umphress, 2019). Concealing a supervisor’s mistakes from the organization and deceiving customers to help a supervisor improve his or her performance are two examples.

Some scholars (Umphress and Bingham, 2011; Johnson and Umphress, 2019; Bryant and Merritt, 2021) argue that UPSB is a specific type of unethical pro-organization behavior (UPOB) and consider UPSB complying with the two key components of the definition of UPOB (Umphress et al., 2010; Umphress and Bingham, 2011): (1) unethical; (2) the intent to promote the effective functioning of the organization. Although Umphress and Bingham (2011) point out that the unethical behavior serving the interests of the organizational members falls into the scope of UPOB, they in fact take the members as the agency to help the organization (Cheng et al., 2021a) and have ignored that the interests of the organization and the supervisor can sometimes conflict with each other (Jensen and Meckling, 1976). Thus, other scholars claim that UPSB is not a simply specific type of UPOB because it can benefit the supervisor while harming the organization at the same time (Cheng and Lin, 2019; Mesdaghinia et al., 2019; Li et al., 2021). Mesdaghinia et al. (2019, p. 493) have directly pointed out that “…[UPSB] serves the interests of the supervisor, sometimes at the expense of the organization.” Similarly, Li et al. (2021, p. 3) argued that “UPSB focuses on its pro-supervisor aspect, yet is perceived as unethical by the larger society and might even be detrimental to the organization. For example, concealing a supervisor’s misconduct of receiving bribes for promotion helps the supervisor avoid punishment, but it violates shareholders’ and the organization’s interests.” These scholars distinguish the intent to benefit the organization and the intent to help the supervisor. It can be inferred that only when employees have the intents to help both of the supervisor and the organization, the scope of UPSB overlaps with that of UPOB. In sum, the key components of the definition of UPSB are unethical and the intent to help the supervisor, and there exists two understandings of UPSB based on whether this behavior beneficial for the organization or not.

Based on the assumption that UPSB indirectly helps the organization by helping its supervisors, some studies have explored how leadership and organization factors influence employees’ UPSB (Johnson and Umphress, 2019; Cheng et al., 2021a). Johnson and Umphress (2019) have identified organizational identification as a key antecedent of UPSB, because those who are highly identified want to benefit the organizations through promoting the effectiveness of the supervisor. However, they largely ignored UPSB benefits supervisors at the expense of the organization’s interests. We might get different findings when we consider this point.

The current study focuses on the UPSB promoting the interests of the supervisor at the expense of the organization and aims to re-examine the relationship between organizational identification and UPSB. Based on social identity theory and social cognitive theory, we propose that organizational identification might decrease UPSB harming the organization because those who are highly identified feel strongly obligated to care about the organization and to achieve its goals.

Moreover, the theories have clearly proposed that employees’ unethical behavior is conditioned by situational factors (Bandura, 1991). Perceived organizational cronyism refers to an employee’s perceptions about the supervisors favoring on employees based their personal relationships rather than performance standards (Turhan, 2014; De Clercq et al., 2021). When employees perceive there exist such phenomena in their organization, they are more willing to take pro-supervisor behaviors (e.g., Shaheen et al., 2019). Considering UPSB is a supervisor-focused behavior and can be used as a strategy to gain the favor of the supervisor (Cheng et al., 2021a), employee’s perceived organizational cronyism is likely to affect the effect of organizational identification on UPSB.

**Figure 1:**
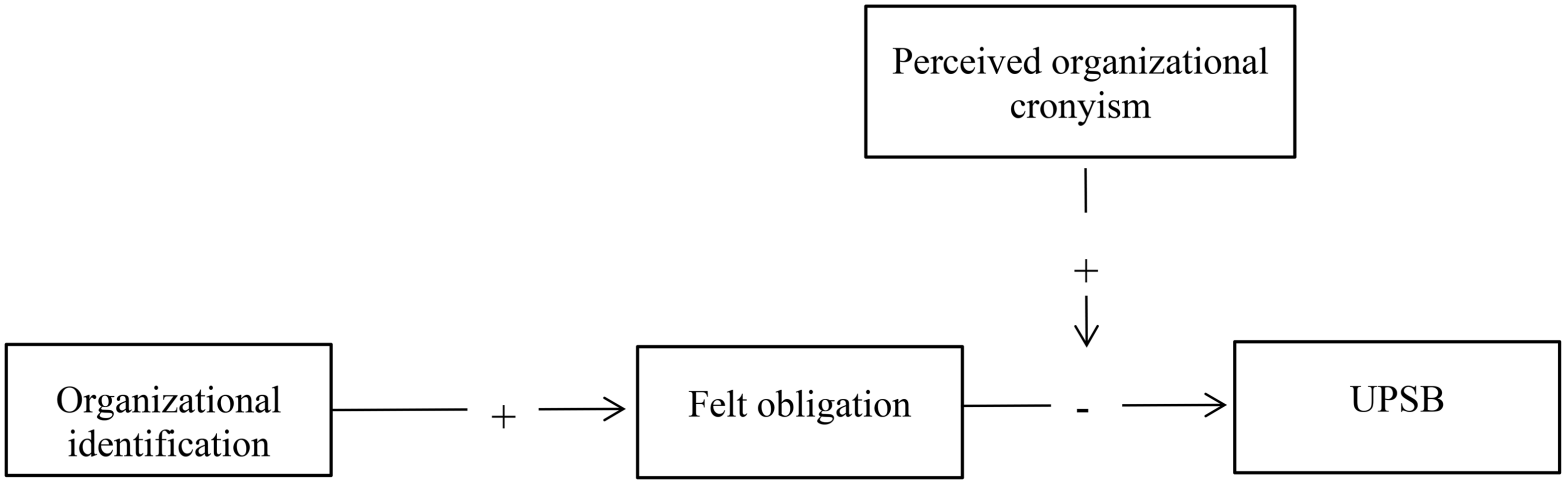
Framework model.

This study makes the following contributions: First, this study contributes to the literature about UPSB by providing preliminary evidence that there exist different kinds of UPSB. We find that organizational identification negatively affects UPSB harming the organization is opposite to the finding by Johnson and Umphress (2019) with the understanding that UPSB indirectly helps the organization, which illustrates the likely existence of the two kinds of UPSB based on whether this behavior beneficiary for the organization or not. Second, the study reveals a new mechanism: felt obligation, thus deepening our understanding of why organizational identification matters. Last, the study explores the moderating effects of perceived organizational cronyism on the negative influence of organizational identification on UPSB, which helps us better understand what context might breed more UPSB. Figure 1 presents the moderated mediation model underlying our research.

## 2. Theoretical background and hypotheses development

### 2.1. Organizational identification and UPSB

Organizational identification refers to the extent to which individuals define themselves as members of an organization (Mael and Ashforth, 1992). Individuals with a high level of organizational identification are more inclined to exhibit beneficial behaviors and contribute to the organization’s success (Haslam and Ellemers, 2005), even at the expense of others’ interests. For example, some scholars found that a high level of organizational identification could make employees engage in unethical behaviors that improve the organization’s efficiency at the expense of the customers’ interests (Umphress et al., 2010). This effect has received much empirical support (Umphress et al., 2010; Effelsberg et al., 2014; Kalshoven et al., 2016; Kong, 2016; Baur et al., 2019).

Specifically, Johnson and Umphress (2019) found that organizational identification could increase individuals’ UPSB, which in turn promotes the organization’s interests. However, their research considers UPSB as a way to indirectly help the organization and ignored the potentially harmful effect of UPSB on the organization (Mesdaghinia et al., 2019; Li et al., 2021). In an organization, it is common that there exist conflicts of interest between the supervisor and the organization (Jensen and Meckling, 1976). For example, if employees help a supervisor to cover up mistakes, the organization will lose an opportunity to correct such errors, which will ultimately result in losses for the organization. This study focuses on the aspect of UPSB that benefits supervisors but harms organizations (Mesdaghinia et al., 2019; Li et al., 2021).

According to social identity theory, with high organizational identification, employees are more inclined to define themselves based on their organizations and regard their organizations’ goals as their own (Mael and Ashforth, 1992). Such self-definition would motivate people to carry out activities that support and protect the interests of the organization (Ashforth and Mael, 1989). Therefore, they are less likely to engage in behavior that could be detrimental to the interests of the organization (Tajfel and Turner, 1986). UPSB increases supervisors’ benefits at the expense of the organization. As the level of organizational identification of employees increases, employees would care more about the organizations’ benefits and behave in line with the role requirements of the organization (Hekman et al., 2009), which will make them less willing to display UPSB. Thus, we propose:

*Hypothesis 1 (H1)*: Organizational identification negatively affects UPSB.

### 2.2. The mediating role of employees’ felt obligation

According to social identity theory, when individuals define themselves as an organizational member, the self-concept will make individuals realize a stereotypical role mode as an organizational member about “what one should think and feel, and how one should behave” (Hogg et al., 1995, p. 260; Ashforth et al., 2008). When a certain identity is salient in mind, the stereotypical role mode of this identity is more internalized into this individual, which leads this individual to better understand and undertake the obligations and responsibilities brought by this identity (e.g., Hekman et al., 2009).

In an organization, employees’ felt obligation is the belief held by individual employees that they should care for the healthy development of the organization and achieve its goals (Eisenberger et al., 2001), which reflects an employee’s understanding of the obligations that he or she should assume as a member of the organization. With the increase of employees’ organizational identification, employees increasingly regard organizational membership as the core identity to define their self-concept, which makes employees more clear about the obligations and responsibilities that organizational members need to undertake, and more clear that the obligations that organizational members need to perform are to safeguard and promote the interests of the organization, and regard the goals of the organization as their own goals. In other words, organizational identification will affect the formation of employees’ felt obligation.

When employees feel obligated to their organizations, they are motivated to achieve the group’s goals and ensure their benefits (Thompson et al., 2020; Cheng et al., 2021b). If employees fail to fulfill their obligations, they feel guilty and this threatens their self-concept (e.g., Wang et al., 2021). To avoid this sense of guilt and threat to their self-concept, individuals with high felt obligation will be more inclined to display positive attitudes toward their organizations and conduct pro-organizational behaviors (Roch et al., 2019). For example, felt obligation was found to promote organizational citizenship behavior (Thompson et al., 2020) and inhibit unethical pro-family behavior (Cheng et al., 2021b). UPSB is a type of unethical behavior that improves the interests of supervisors by harming the interests of the organization, which is contrary to the obligations that employees should fulfill as members of the organization. The stronger their obligations, the less likely they are to engage in such behavior. Therefore, the study proposes the following hypothesis:

*Hypothesis 2 (H2)*: The negative effect of organizational identification on UPSB is mediated by employees’ felt obligation.

### 2.3. The moderating effect of perceived organizational cronyism

According to social cognitive theory, the ethical behaviors of employees are controlled by their self-regulation mechanism, which is highly contextualized—whether it is activated or not is affected by the individual’s cognition at the time and the individual’s recognition and interpretation of the situational factors (Bandura, 1991). When an employee’s intended behavior violates his/her own self standards, the employee will self-condemn the expected unethical behavior, and then control himself/herself not to conduct the behavior (Bandura, 1991). However, when employees can find adequate reasonable excuses for their unethical behaviors, their self-regulation mechanism can be dysfunctional, so that they can avoid self-condemnation when implementing unethical behaviors and conduct unethical behavior without psychological burden (Bandura, 1999).

With the increase of employees’ felt obligation, employees become more aware of their obligations to care for the organization and achieve its goals as a member of the organization (Eisenberger et al., 2001). If an employee fails to fulfill his/her obligations, he/she will feel guilty and threaten his/her self-concept (e.g., Wang et al., 2021). Therefore, employees with higher sense of obligation are more likely to recognize the destructiveness of UPSB to the organization, recognize the unethical nature of the behavior, and activate the self-regulation mechanism to inhibit the occurrence of the behavior.

Organizational cronyism refers to the phenomenon that supervisors tend to show favoritism toward certain subordinates and prioritize their interests based on the quality of personal relationships rather than performance-based standard (Turhan, 2014). When employees perceive a high level of organizational cronyism, they will believe that loyalty to leaders is the most important criterion for evaluating the worthiness of an organizational member in the eyes of their supervisors (Turhan, 2014). The general emphasis on loyalty to their direct supervisors will make employees more likely to consider UPSB as reasonable behavior to express “loyalty” in the organization (Turhan, 2014). In addition, considering supervisors are one of key factors for employees’ career success (e.g., Akkaya et al., 2022), employees might find that some employees in the organization receive benefits because they show loyalty and obedience to the supervisors, which makes employees eager to get the same preferential treatment (Shaheen et al., 2019). Taken together, when employees perceive high level of organizational cronyism, they are easier to find acceptable excuses to rationalize their UPSB and thus their self-regulation mechanism is harder to be activated by felt obligation to curb their UPSB. As a result, they are more likely to exhibit UPSB. On the contrary, when employee’s perceived organizational cronyism is of low level, people might withhold UPSB because their self-regulation mechanisms more likely to be activated by felt obligation due to the lack of enough excuses for their UPSB. Therefore, the study proposes the following hypothesis:

*Hypothesis 3 (H3)*: Employees perceived organizational cronyism will moderate the effect of employees’ felt obligation on UPSB. Specifically, as the level of perceived organizational cronyism increases, the negative effect of employees’ felt obligation on UPSB will be weakened.

Furthermore, combining H2 and H3, based on social identity theory and social cognitive theory, this study argues that the mediating effect of employee’s felt obligation between organizational identification and UPSB is affected by the perceived organizational cronyism. With the increase of organizational identification, employees are more likely to form felt obligation due to the salience of the identity as organizational membership, which makes it easier for employees to identify the damage of UPSB to the organization, thus activating the self-regulation mechanism and inhibiting their own UPSB. When employees have a high level of perceived organizational cronyism, they will think that giving priority to the interests of supervisors and showing their loyalty to supervisors are universal behaviors in the organization, and thus they are more likely to think that UPSB is a reasonable “loyalty” behavior in the organization. As a result, the self-regulation mechanism activated by the sense of obligation is inhibited, so that they can exercise UPSB without psychological burden. On the contrary, when employees have a low level of perceived organizational cronyism, the self-regulation mechanism activated by employees based on felt obligation will normally play a role in inhibiting UPSB.

Combining H2 and H3, this study proposes the following hypothesis:

*Hypothesis 4 (H4)*: Employees perceived organizational cronyism will moderate the mediating effect of employees’ felt obligation between organizational identification and UPSB. Specifically, as the level of perceived organizational cronyism increases, the negative impact of organizational identification on UPSB through employees’ felt obligation diminishes.

## 3. Participants and study design

### 3.1. Data collection

Since UPSB was found to be popular in many industries and organizations (Mesdaghinia et al., 2019), we intended to collect a sample of adults with full-time jobs from different industries. In this study, we collected data from a database platform^1^ where there are millions of online respondents from multiple industries. The quality of data collected from this platform has been demonstrated by many papers published in top-ranked journals (e.g., Huang and Sengupta, 2020; Chang et al., 2022).

To reduce common method bias, we conducted a three-wave questionnaire-based survey with a time lag of 2 weeks. We restricted the range of potential participants to adults with full-time jobs in government, enterprises, or public institutions. To avoid that one participant takes multiple surveys at one time, we required each IP address to be limited to completing the questionnaire once at every stage. In each round of the questionnaire survey, attention-check questions were added to ensure data quality.

Questionnaires were distributed to 806 employees, and 582 employees participated in three consecutive surveys. The overall employee retention rate of the three surveys was 72.21%. After deleting the questionnaires of four employees who had either responded in a perfunctory way or failed the attention check test, 578 valid questionnaires were obtained. In the final sample, men accounted for 44.6% of the total, and women for 55.4%. The sample’s average age was 29.94 years old (*SD =* 6.11), with 61.1% under 30 years old, 32.8% between 30 and 40 years old, and 6.1% over 40 years old. In terms of education level, 3.8% had completed general high school/technical secondary school/technical school/vocational high school, 13.1% had completed junior college, 73.9% had a bachelor’s degree, 8.7% a master’s degree, and 0.5% a doctoral degree. The average job tenure was 5.25 years (*SD =* 4.96), and the average tenure with the direct supervisor was 2.93 years (*SD =* 2.29). In terms of the type of organizations, 10.7% were government agencies, 12.3% were public institutions, 50.7% were private enterprises, 21.5% were state-owned enterprises, and 4.8% were foreign-funded enterprises.

### 3.2. Measures

The scales used in this study are all mature scales published in top-tier journals and widely accepted by scholars in the field of organizational behavior. The researchers followed a strict “translation and back-translation” procedure (Brislin, 1970) to translate the original English scale into Chinese.

#### 3.2.1. Organizational identification

In this study, the six-item scale of Mael and Ashforth (1992) was used to measure organizational identification (*α* = 0.83).

#### 3.2.2. Employees’ felt obligation

The seven-item scale of Eisenberger et al. (2001) was used to measure felt obligation. (*α* = 0.79).

#### 3.2.3. Perceived organizational cronyism.

This study used the 15-item scale developed by Turhan (2014) to measure perceived organizational cronyism (six items measured insider preferences, five items measured paternalistic cronyism, and four items measured preferences based on reciprocal exchange relationships; *α* = 0.90).

#### 3.2.4. Unethical pro-supervisor behavior

Since there is no mature scale for measuring the type of UPSB that harms the interests of the organization, this study adopted the scale of Johnson and Umphress (2019) but changed the reference from “others” to “organization” and defined supervisor as the “direct supervisor.” The scale contains six items: “Because it was necessary, I concealed information from the organization that could be damaging to my supervisor,” “Because my direct supervisor needed me to, I did not reveal to other members of the organization a mistake my supervisor made that would damage the supervisor’s reputation,” “Because it helped my supervisor, I exaggerated the truth about my supervisor’s performance to the organization,” “Because it benefited my supervisor, I withheld negative information about my supervisor’s performance from others in the organization,” “Because it helped my supervisor, I misrepresented the truth to make my supervisor look good,” and “Because my supervisor needed me to, I spoke poorly of another individual in the organization who was a problem for my supervisor”.

We conducted a pilot test to examine the reliability and validity of the scale. The questionnaire included items measuring UPSB and other concepts similar to UPSB that could show supervisor-centered behavior (e.g., supervisor-directed citizenship behavior, supervisor-directed ingratiation, and political behavior). The scale of Liao and Rupp (2005) was used to measure supervisor-directed citizenship behavior, the scale developed by Ingold et al. (2015) was used to measure supervisor-directed ingratiation, and the scale developed by Gabriel et al. (2018) was used to measure political behavior. Results of the pilot study including 200 employees showed that the internal consistency coefficient α of the UPSB scale was 0.92 and this scale had good discriminant validity with other similar constructs. Full results could be obtained from the authors.

#### 3.2.5. Control variables

As the job tenure and the tenure with the direct supervisor could affect employees’ cognition and attitude toward the organization and supervisor, the research took the two as control variables. Gender, age, educational background, and organization type were also taken as control variables.

## 4. Results

### 4.1. Confirmatory factor analysis

This study used Mplus 8.0 to conduct the confirmatory factor analysis. The details of the analysis results are summarized in Table 1. As perceived organizational cronyism is a three-dimensional structure, we created parcels based on these subdimensions (Little et al., 2002). The results of the analysis show that compared with other competitive models, the four-factor model has the best fit (*χ*^2^ = 514.26; DF = 201; *χ*^2^/DF = 2.56; CFI = 0.95; TLI = 0.94; RMSEA = 0.05; SRMR = 0.05). This indicates good discriminant validity among these four variables.

**Table 1:** Results of the confirmatory factor analysis.

Model	*χ* ^2^	*df*	*χ*^2^/*df*	CFI	TLI	RMSEA	SRMR
Model 1: A, B, C, D	514.26	201.00	2.56	0.95	0.94	0.05	0.05
Model 2: A + B, C, D,	952.38	204.00	4.67	0.88	0.86	0.08	0.07
Model 3: A + B + C, D	1388.29	206.00	6.74	0.81	0.79	0.10	0.09
Model 4: A + B + C + D	3368.42	207.00	16.27	0.49	0.43	0.16	0.18

*N* = 578. A = perceived organizational cronyism, B = felt obligation, C = organizational identification, D = UPSB.

### 4.2. Descriptive analysis

The mean, standard deviation, and correlation coefficients of all the study’s variables are shown in Table 2.

**Table 2:** Variable mean, standard deviation, and correlation coefficient.

	*M*	SD	1	2	3	4	5	6	7	8	9	10
1 Age	29.94	6.11	–									
2 Gender	0.55	0.50	−0.05	–								
3 Education	2.06	0.24	−0.08	0.05	–							
4 Job tenure	5.35	4.96	0.82^**^	−0.03	−0.0.10^*^	–						
5 Tenure with the direct supervisor	2.93	2.29	0.52^**^	−0.02	−0.05	0.60^**^	–					
6 Organization type	2.27	1.09	−0.22^**^	0.02	−0.18^**^	−0.25^**^	−0.12^**^	–				
7 Organizational identification (T1)	5.50	0.87	0.18^**^	−0.07	0.04	0.21^**^	0.20^**^	−0.10^*^	0.45			
8 Felt obligation (T2)	5.31	0.80	0.24^**^	−0.06	0.05	0.26^**^	0.28^**^	−0.06	0.51^**^	0.55		
9 Perceived organizational cronyism (T2)	3.86	0.50	−0.06	−0.02	0.03	−0.05	−0.12^**^	−0.08	−0.20^**^	0.67^**^	0.51	
10 UPSB (T3)	3.17	1.28	−0.09^*^	−0.09^*^	0.05	−0.10^*^	−0.17^**^	0.07	−0.14^**^	0.33^***^	0.39^***^	0.64

*N* = 578; ^*^p < 0.05, ^**^p < 0.01, ^***^p < 0.001; Gender: 0 = male, 1 = female; Education: 0 = general high school/technical secondary school/technical school/vocational high school, 1 = junior college, 2 = bachelor’s degree, 3 = master’s degree, 4 = doctoral degree; Type of organization: 0 = government, 1 = public institution, 2 = state-owned enterprise, 3 = private enterprise, 4 = foreign enterprise; average variance extracted (AVE) for constructs are provided on the diagonal.

### 4.3. Convergent and discriminant validity

We checked the convergent and discriminant validity among constructs. Given that all average variance extracted (AVE) values were bigger than the squared correlation between a specific variable and any other variables (see Table 2), and most of AVE were bigger than 0.5, the measurement model had acceptable convergent validity and discriminant validity.

### 4.4. Hypothesis tests

#### 4.4.1. The negative influence of organizational identification on UPSB

After controlling employees’ age, gender, educational background, job tenure, tenure with the direct supervisor, and organization type, we took *organizational identification* as the independent variable and *UPSB* as the dependent variable to conduct a hierarchical regression. Table 3 summarizes the results. Model 4 shows that organizational identification has a significant negative effect on UPSB (*B* = –0.12; *p* = 0.006 < 0.01). Therefore, H1 is supported.

**Table 3:** The test of the mediating effect of employees’ felt obligation by causal stepwise regression.

Variable	Employees’ felt obligation				UPSB					
	Model 1		Model 2		Model 3		Model 4		Model 5	
	*b*	SE	*b*	SE	*b*	SE	*b*	SE	*b*	SE
Control variable										
Age	0.07	0.01	0.07	0.01	0.00	0.02	0.00	0.02	0.02	0.01
Gender	−0.06	0.06	−0.03	0.06	−0.10^*^	0.11	−0.11	0.11	−0.12^**^	0.10
Education	0.08^*^	0.05	0.05	0.05	0.06	0.09	0.06	0.09	0.08	0.09
Job tenure	0.09	0.01	0.04	0.01	0.02	0.02	0.04	0.02	0.05	0.02
Tenure with the direct supervisor	0.20^***^	0.02	0.14^**^	0.02	−0.18^**^	0.03	−0.16^**^	0.03	−0.12^**^	0.03
Organization type	0.01	0.03	0.03	0.03	0.06	0.05	0.06	0.05	0.07	0.05
Independent variables										
Organizational identification			0.46^**^	0.03			−0.12^**^	0.06	0.02	0.07
Mediating variable										
Employees’ felt obligation									−0.30^***^	0.08
*R* ^2^	0.10		0.30		0.04		0.06		0.12	
Δ*R*^2^			0.29^***^				0.05^***^		0.10^***^	

*N* = 578; ^*^p < 0.05, ^**^p < 0.01, ^***^p < 0.001.

#### 4.4.2. The mediating effect of employees’ felt obligation

To test the mediating effect of employees’ felt obligation, we used Baron and Kenny’s (1986) method. The results are summarized in Table 3. Model 2 shows that the independent variable *organizational identification* has a significant positive effect on the mediating variable *felt obligation* (*B* = 0.46; *p* = 0.000 < 0.001). Model 5 shows that employees’ felt obligation has a significant negative effect on UPSB (*B* = –0.30; *p* = 0.000 < 0.001), and the effect of organizational identification on UPSB is not significant (*B* = 0.02, *p* = 0.640 > 0.05). We also employed bootstrapping methods to retest the mediating effect. Results reveal there is a nonsignificant direct effect, *B* = 0.03, 95%CI = [−0.101,0.165], and a significant indirect effect, *B* = –0.21, 95%CI = [−0.288, −0.126]. Therefore, felt obligation fully mediate the relationship between organizational identification and UPSB. Thus, H2 is supported.

#### 4.4.3. The moderating effect of perceived organizational cronyism

Next, we used hierarchical regression to test the moderating effect of perceived organizational cronyism. Before entering the variables into the regressions, we centralized the mediating variable *felt obligation*, and the moderating variable *perceived organizational cronyism*. After controlling *age*, *gender,* and other variables, the results are summarized in Table 4.

**Table 4:** Moderating effect of organizational cronyism.

Variable	Dependent variable: UPSB Moderator: Perceived organizational cronyism							
	Model 1		Model 2		Model 3		Model 4	
	*b*	SE	*b*	SE	*b*	SE	*b*	SE
Control variable								
Age	0.00	0.02	0.02	0.01	0.02	0.01	0.04	0.01
Gender	−0.10^**^	0.11	−0.12^**^	0.10	−0.12^**^	0.10	−0.12^***^	0.10
Education	0.06	0.09	0.08	0.08	0.07	0.08	0.07	0.08
Job tenure	0.02	0.02	0.05	0.02	0.03	0.02	0.01	0.02
Tenure with the direct supervisor	−0.18^**^	0.03	−0.12^**^	0.03	−0.10^**^	0.03	−0.10^**^	0.03
Organization type	0.06	0.05	0.07	0.05	0.10	0.05	0.08	0.05
Mediating variable								
Employees’ felt obligation			−0.29^***^	0.07	−0.16^***^	0.07	−0.22^***^	0.07
Moderating variable								
Perceived organizational cronyism					0.32^***^	0.06	0.27^***^	0.06
Interaction term								
Employees’ felt obligation × perceived organizational cronyism							0.15^***^	0.06
*R* ^2^	0.03		0.11		0.20		0.22	
Δ*R*^2^			0.08^***^		0.09^***^		0.02^***^	

*N* = 578; ^*^p < 0.05, ^**^p < 0.01, ^***^p < 0.001.

**Figure 2:**
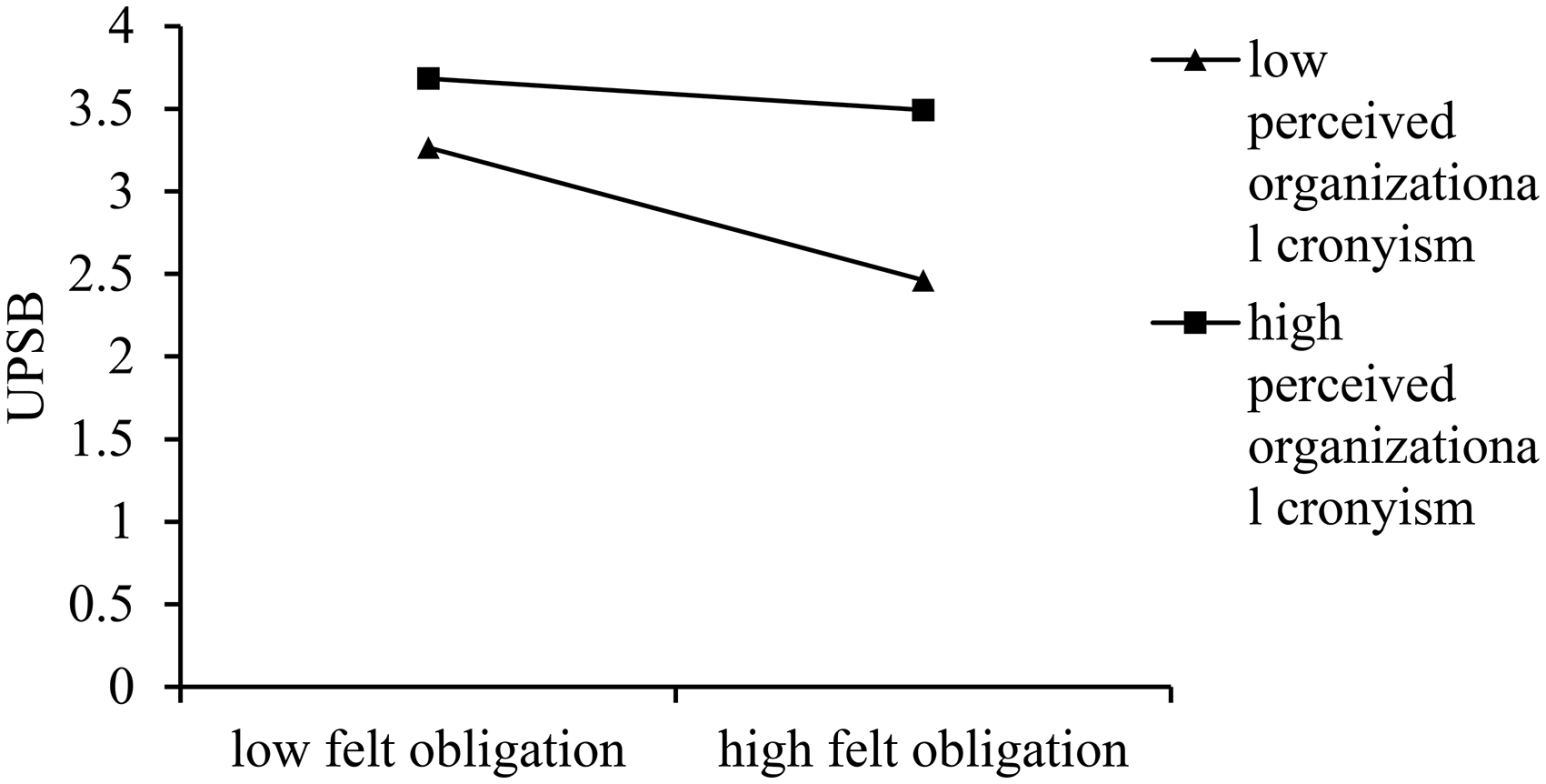
The moderating effect of perceived organizational cronyism.

Model 4 in Table 4 shows that the interaction term of felt obligation and perceived organizational cronyism has a significant positive effect on UPSB (*B* = 0.15; *p* = 0.000 < 0.001). We then calculated simple effects to better illustrate the moderating effect. The results are shown in Figure 2. The negative effect of felt obligation on UPSB is nonsignificant when employees perceive a high level of organizational cronyism (*B* = –0.13; *SE* = 0.07; 95% CI = [−0.279, 0.01]), and significant when employees perceive a low level of organizational cronyism (*B* = –0.56; *SE* = 0.10; 95% CI = [−0.766, −0.359]). Hence, H3 is supported.

#### 4.4.4. The moderated mediation effect

To test H4, based on the macro-process model 14 of SPSS, the bootstrapping method (5,000 replications) is used to test the second stage moderated mediation. The results show that when employees perceive a high level of organizational cronyism, the indirect effect of organizational identification on UPSB is not significant (indirect effect = −0.07; *SE* = 0.04; 95% CI = [−0.155, 0.013]). When employees perceive a low level of organizational cronyism, the indirect effect of organizational identification on UPSB is significant (indirect effect = −0.26; *SE* = 0.06; 95% CI = [−0.384, −0.136]). In addition, the index of moderated mediation is 0.09 (*SE* = 0.03; 95% CI = [0.030, 0.162]). Thus, H4 is supported.

## 5. Discussion

In the current study, based on social identity theory, we proposed a moderated mediation model linking organizational identification and UPSB. Based on a three-wave design, we found a negative association between these two variables. Besides, we showed the mediation effect of felt obligation and the moderation effects of perceived organizational cronyism. These results carry important implications.

### 5.1. Theoretical implications, and contributions

First of all, this study re-examines the influence of organizational identification on UPSB and finds a negative association between organizational identification on UPSB, which is different from previous studies (Johnson and Umphress, 2019). This finding showed that previous studies ignored an important point: UPSB might harm organizations. These studies examined the influence on UPSB by merely emphasizing that UPSB could indirectly promote organizational interests by helping leaders (Johnson and Umphress, 2019; Bryant and Merritt, 2021). This study shows that the research about UPSB is incomplete if it ignored the damage UPSB might have on an organization when exploring the influence of employees’ relationships with the organization on the UPSB. In addition, this study contributes to the UPSB literature by providing preliminary evidence that there exist different types of UPSB based on whether this behavior beneficial for the organization or not.

Second, the study reveals the mediating effect of felt obligation. So far, this is the first study to reveal the mechanism through which organizational identification influences UPSB. This study adds to the literature, and found that identification might increase people’s obligation to their organizations, which would inhibit UPSB. This study deepens our understanding of why organizational identification could reduce UPSB.

Last, this study reveals the boundary conditions of organizational identification affecting UPSB. We found that perceived organizational cronyism could buffer the effect of organizational identification. These results showed that UPSB is a person-in-situation phenomenon. That is, while people would make decisions based on their identification and felt obligations, their final behavior was still influenced by their perceptions about the situation, which is in line with the prior research that employees’ behavior is highly influenced by the situation (e.g., Haider et al., 2022).

### 5.2. Practical implications

This study has the following implications for management practice. First, organizations need to be aware of the positive effect of organizational identification and guide employees to establish the correct value orientation. Organizations could make it clear to managers that employees’ organizational identification can increase their felt obligation to care for the organization, and thus reduce non-ethical behaviors for supervisors that disregard the interests of the organization. Managers should utilize the positive side of organizational identification, make employees take their organization to heart, always take the interests of the organization into account, and emphasize the overall interests of the organization as a priority.

Second, organizations should create a favorable working environment and stick to meritocracy and oppose favoritism. This study reveals that in organizations where nepotism is prevalent, employees are more likely to engage in unethical supervisor-centered behavior. As managers are key to creating working environment (e.g., Tanveer et al., 2013; Jiang et al., 2022), managers should adhere to the principle of merit-based selection, explore, and select excellent talents with objective ability, and rationally allocate and use these talents. This requires managers to not judge employees according to their likes and dislikes, close relationships, or personal grudges, and to not encourage cliques. Instead, managers should realistically evaluate the ability and potential of each employee. When a healthy and harmonious atmosphere is created in the organization and unethical ways of selecting and employing people are eliminated, organizations are in a better position to promote cohesion, creativity, and increase effectiveness.

### 5.3. Limitations and future directions

Although this paper makes a theoretical contribution to the field of UPSB research and has implications for management practice, there are a few limitations, which might encourage further improvement and the development of related research in this field.

First, this study focuses on UPSB that causes harm to an organization and ignores UPSB that indirectly benefits an organization. Future research could systematically theorize the two types of UPSB, create corresponding scales of them and investigate the different effects of various antecedents on them to advance our understanding of their conceptual scopes.

Second, while the study investigated the role of perceptions of situation in the process of displaying UPSB, it did not investigate the boundary conditions at an organizational and team level. Cross-level research could extend our understandings of the causes of UPSB at the organizational and team levels. To fill this gap in the literature, we encourage future research on the antecedents and boundary effects at the organizational and team levels.

Third, this study did not consider the differences of employees from various generations. For example, as employees of Generation Z born in the Internet age are natives of self-motivators (Dobrowolski et al., 2022), they may exhibit different tendencies for UPSB compared with other generations. Thus, future research can explore how different generations exhibit different tendencies to exhibit UPSB.

Last, this study was primarily conducted in China. Previous research has found that in East Asian countries that value harmony, leaders are placed at more importance places than organizations (Turhan, 2014). Our hypotheses have been verified in Chinese organizations, but more research needs to be conducted on whether the same conclusion can be drawn in the West.

## Data availability statement

The raw data supporting the conclusions of this article will be made available by the authors, without undue reservation.

## Ethics statement

Ethical review and approval was not required for the study on human participants in accordance with the local legislation and institutional requirements. The patients/participants provided their written informed consent to participate in this study.

## Author contributions

TS was responsible for conceptualization, methodology, investigation, formal Analysis, and the original draft writing. WS was in charge of data curation. JW reviewed and edited the manuscript. All authors contributed to the article and approved the submitted version.

## Conflict of interest

TS was employed by the company Industrial and Commercial Bank of China.

The remaining authors declare that the research was conducted in the absence of any commercial or financial relationships that could be construed as a potential conflict of interest.

## Publisher’s note

All claims expressed in this article are solely those of the authors and do not necessarily represent those of their affiliated organizations, or those of the publisher, the editors and the reviewers. Any product that may be evaluated in this article, or claim that may be made by its manufacturer, is not guaranteed or endorsed by the publisher.

## Footnotes

^1^ https://www.credamo.com
